# Protein quality, nutrition and health

**DOI:** 10.3389/fnut.2024.1406618

**Published:** 2024-05-28

**Authors:** Juliane Calvez, Dalila Azzout-Marniche, Daniel Tomé

**Affiliations:** AgroParisTech, INRAE, Université Paris-Saclay, Paris, France

**Keywords:** nutrition, protein for human health, protein quality, protein, amino acids

## Abstract

Dietary proteins are energy macronutrients providing nitrogen, amino acids (AA), and energy. AAs are the main nitrogen-containing compounds in the body and are the precursors for the synthesis of body proteins and of several other AA-derived molecules. Among the 20 AAs included in protein sequence, 9 are classified as “nutritionally essential” or “indispensable” AA (IAA) because they cannot be synthesized in the body and must be provided by the diet. IAAs are limiting components for protein synthesis. An adequate intake of protein is required to support growth, maintenance, body functions, health and survival. Official definition of protein requirement is based on nitrogen balance. Protein quality is related to the capacity of protein to provide an adequate quantity of nitrogen and of each of the 9 IAAs for the different physiological situations in humans. Protein source is considered high quality for humans when the protein is readily digested, simultaneously providing an adequate quantity of nitrogen and of each of the 9 IAAs to maintain an adequate metabolic AA pool. The most accurate assessment of protein quality of foods for humans is through metabolic studies that measure nitrogen balance. The protein quality score is the ratio of the content of each IAA in the food and in a reference profile. This score corresponds to the calculated composition of a protein which, when meeting protein requirements, simultaneously meets the requirements of each of the 9 IAAs. AA scores as predictors of protein quality must be adjusted for protein and AA availability.

## 1 Introduction

Dietary proteins are macronutrients providing nitrogen, amino acids (AAs), and energy. In living organisms, nitrogen is mostly associated to AAs and AAs are mostly in the form of proteins. AAs are the main nitrogen-containing compounds in the body and are the precursors for the synthesis of body proteins and of several other AA-derived molecules, all involved in the structure of tissues and/or in all the functions of the organism.

**Figure 1:**
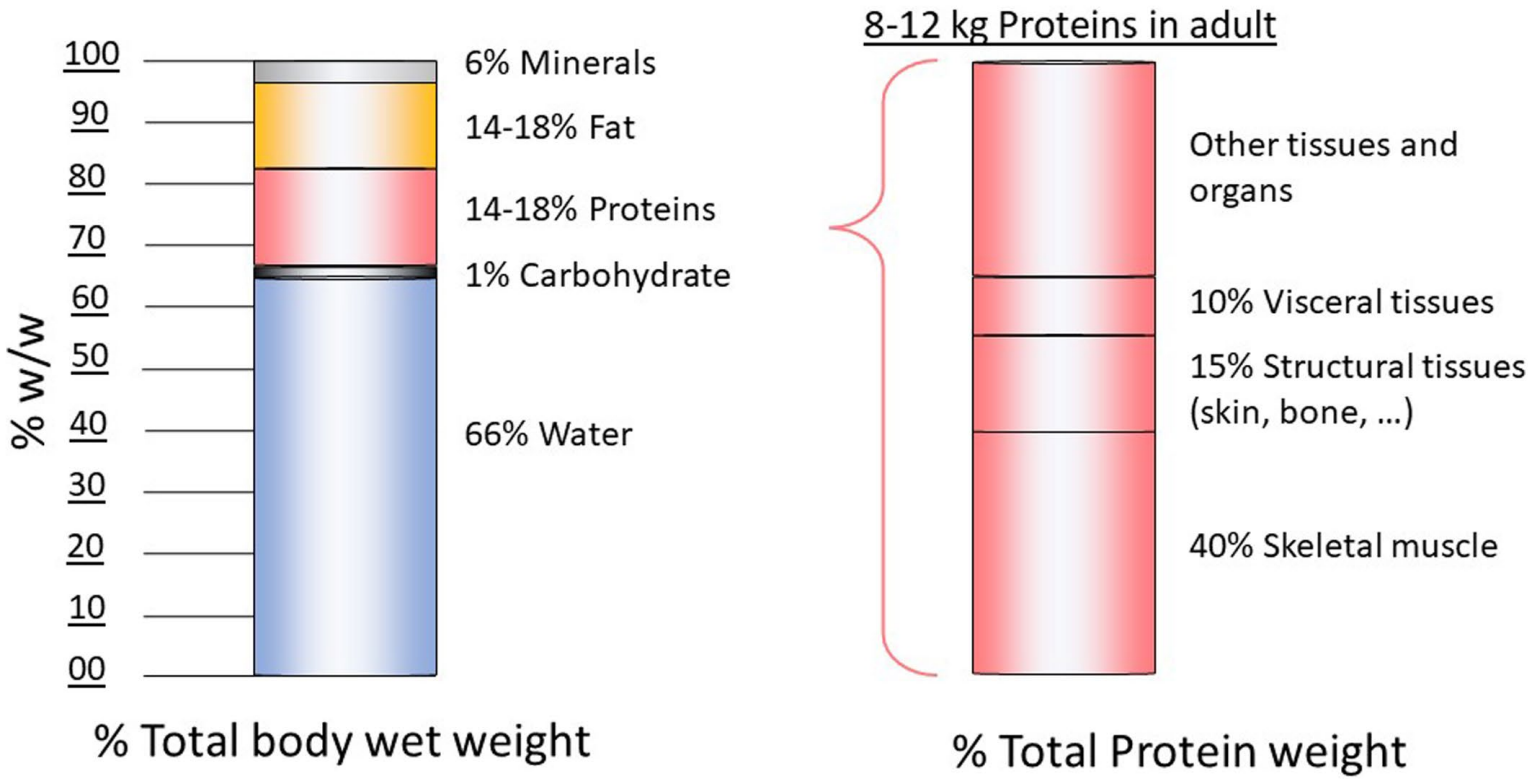
Body composition in healthy adult.

There is a very large number of proteins in the body (~10,000 types) and each protein is characterized by a specific sequence of AAs encoded in the genetic code. Among the 20 AAs included in protein sequence, 9 are classified as “nutritionally essential” or “indispensable” AAs (IAAs) because they cannot be synthesized in the body and must be provided by the diet (1). The 11 other AAs are “dispensable” because they can be synthesized in the body from precursors available in the organism. In adult humans (female 57 kg, male 70 kg), the protein compartment is 8–12 kg (Figure 1). Despite the large number of body’s proteins in the body, about half of these proteins are represented by four proteins—myosin, actin, collagen, and hemoglobin—and among them, about 25% is represented by collagen. Body protein have both structural (muscle, skin, and bone) and physiological function (enzymes, hormones, receptors, antibodies, and cytokines).

Several health outcomes are associated with protein sufficiency such as body weight, body composition, muscle mass and strength, bone health, immune defenses, and most if not all physiological functions. An adequate intake of protein is required to support growth, maintenance, body functions, health, and survival. Protein quality is related to the capacity of protein to provide an adequate quantity of nitrogen and of each of the 9 IAAs for the different physiological situations in humans (1). The nutritive value of proteins from food and diet depends both on the amount of protein provided, but also on the AA composition and concentration, and on the bioavailability of protein-derived nitrogen and AAs. Protein quality matters because there are differences between the different food sources. Moreover, some forms of food storage and processing can affect protein quality (2).

**Figure 2:**
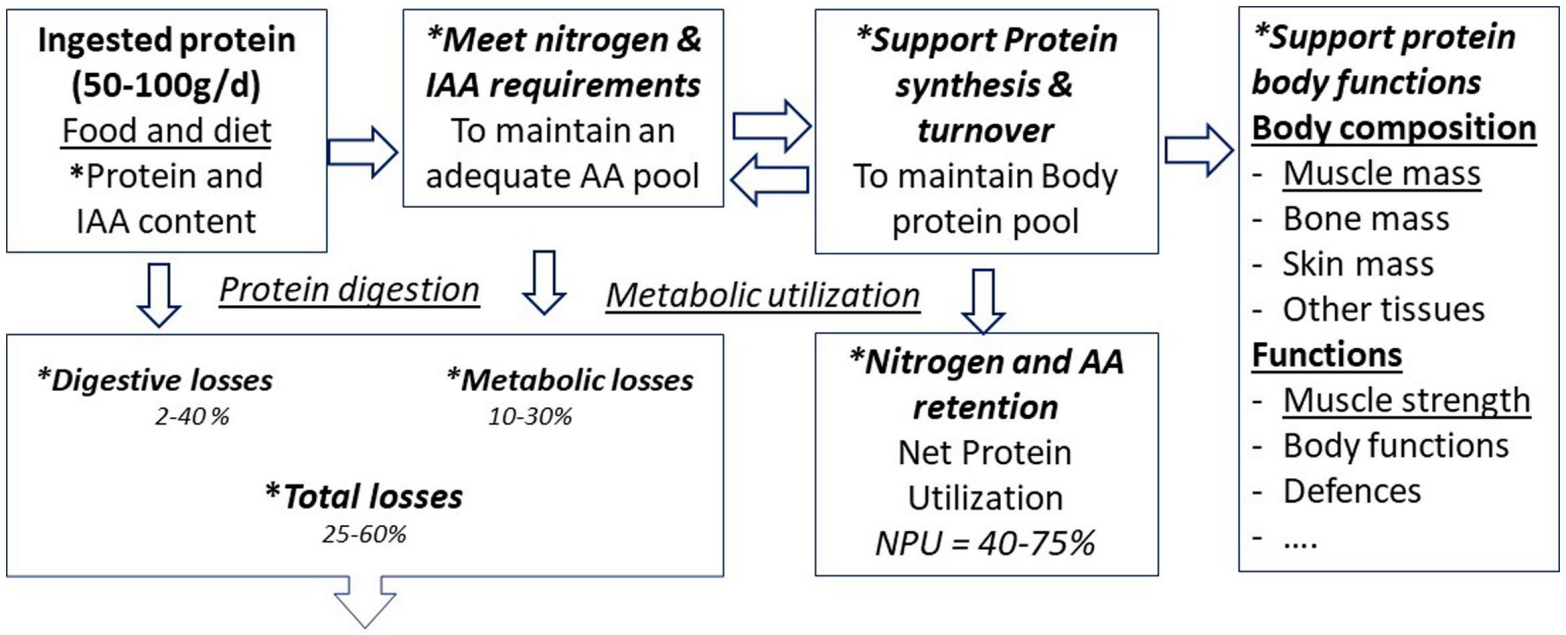
Proteinintake and utilization of nitrogen and amino acids (^*^markers of protein needs and protein quality) and markers and method for assessing protein quality.

Suitable markers for measuring the need for AAs and proteins and protein quality are derived from the different levels of AA metabolism and utilization in the body and from the functions of protein in the body (Figure 2; Table 1). Since the 1970/80s, the priority for international authorities of the United Nations (FAO/WHO/UNU) has been to define the requirement for nitrogen and IAA as criteria for protein quality to support body protein synthesis (1, 3, 4).

**Table 1:** Markers and methods for assessing protein quality.

**Protein, nitrogen & IAA requirements**		
Protein requirement	Nitrogen balance	Digestive and metabolic nitrogen losses
Amino acid requirement	Amino acid oxidation	Stable isotopes amino acid balance
**Protein, nitrogen & AA metabolic fate**		
Protein, nitrogen & amino acid bioavailability	Faecal/ileal digestibility Amino acid availability	Faecal/ileal losses, dual isotope, Indicator AA oxidation, *In vitro*/*in silico* methods
Net protein utilization	Nitrogen/AA retention	Nitrogen/AA losses (stable isotopes)
Protein turnover	Whole body Protein synthesis	Stable isotopes amino acid balance and fluxes
	Muscle protein synthesis	Stable isotopes amino acid administration and muscle tissue sampling
**Protein body functions**		
Body composition	Lean mass, muscle mass, bone mass, …	
Body functions	Muscle strength, defenses, various functions, …	

## 2 Protein and nitrogen requirements

Meeting protein nitrogen needs is required to maintain the body’s protein pool that affect body composition and many if not all the functions in the body. Official definition of protein requirement is based on nitrogen balance – the usual protein intake that maintains a nitrogen balance in a person in good health, with normal body composition, normal energy balance and moderate physical activity. Determined by the nitrogen balance method in adult, the mean protein requirement is 0.66 g protein/kg/d (~40–50 g/d) and the recommended protein intake 0.83 g/kg/d (~50–60 g/d) (1). In different physiological situations such as infants, children, adolescents, pregnant women and lactating women, protein needs are derived from a factorial approach including nitrogen balance and additional protein deposition required for growth, pregnancy, or lactation.

Protein concentration or density (i.e., the amount of protein per unit of food) is a factor of food’s protein quality (5, 6). Measuring nitrogen content with the Kjeldahl or Dumas methods and using a Nitrogen to Protein Conversion Factor remains the more frequently used approach for protein content in foods (7). The default conversion factor used for a mixture of protein sources is 6.25, corresponding to a nitrogen content of 16%. Specific protein conversion factors range from 5.7 (17.5% nitrogen) to 6.4 (15.6% nitrogen) for the major protein sources in the diet. The protein concentration in different food protein sources shows that animal product protein sources such as meat, milk, eggs, and some animal products are rich in protein with protein content of 30–70% (dry weight, dw). Among vegetables, pulses have the highest protein concentrations, ranging from 20–25% (dw) in most raw beans and peas to 35–38% in soybeans and lupines. Cereal seeds have a protein content of 15–20% (dw). Most nuts and edible seeds contain 8–18% protein (dw). Many oil seeds have 12–20% protein (dw), and the cake that remains after oil extrusion can have as much as 30–40% protein (dw).

## 3 Indispensable amino acid requirement and protein quality score

Maintaining optimal protein status required to provide in the diet a bioavailable form of an adequate quantity of protein with an adequate IAA profile.

The AA composition of proteins is usually calculated as milligrams AA per gram of protein. If they are reported as milligrams AA per gram of nitrogen, they are converted to the protein equivalents by multiplying by specific Nitrogen to Protein Conversion Factor. To calculate the AA content of a combination of food proteins, as in a food based on several protein sources or in a mixed diet, a weighted mean of the published or analytical results of each component should be used.

The protein required to achieve nitrogen balance must be of high quality. Protein quality is based on the capacity to provide an adequate quantity of nitrogen and of each of the 9 IAAs to achieve nitrogen balance and to support both protein turnover, and synthesis of the various AA derived component in the body. The 9 IAAs not synthesized in the body and limiting factors of AA utilization for protein synthesis must be provided at an adequate quantity and profile. IAA requirement for adult was initially determined by nitrogen balance in 1985 and re-evaluated in 2007 based on stable isotopes methods (1). IAA requirements were also determined for younger subjects by a factorial approach. The protein quality score is based on the ratio of the content of each IAA in the food and in a reference profile. The reference profile is the calculated composition of a protein which, when meeting protein requirements, simultaneously meets the requirements of each of the 9 IAAs. From the 2007 re-evaluation of IAA requirements, many foods such as cereals and legumes previously thought to be adequate in their IAA content, could be partially limited, particularly in lysine and Sulphur AA, respectively.

## 4 Correction of the score by digestibility

Protein sources are considered high quality for humans when the protein is readily digested, and nitrogen and AA readily absorbed and simultaneously providing an adequate quantity of nitrogen and of each of the 9 IAAs to maintain an adequate metabolic AA pool. A protein may have a good AA composition relative to the reference profile, but if it is not fully digested and its constituent AAs are not absorbed, its capacity to provide nitrogen and IAAs for human function will diminish.

Not all food proteins are digested, absorbed, and utilized to the same extent because of inherent differences in their source (e.g., inside vegetable cells with indigestible membranes), their physicochemical nature (e.g., protein configuration and AA binding), the presence of food constituents that modify digestion (e.g., dietary fiber, tannins, and other polyphenols), the presence of antinutritional factors that interfere with protein breakdown (e.g., trypsin inhibitors and lectins), and processing conditions that alter the nature or release of AAs (e.g., Maillard reaction and formation of polyAAs and methylmercaptan) (2, 8). Protein nitrogen digestibility values and more recently ileal AA digestibility values of specific foods and well-defined diets may be taken from reliable published data or must be determined, preferably in humans (3). When cost and practicality do not permit metabolic studies in humans to be performed, standardized methods in animal models are used (9). Nevertheless, animal data must be used with caution for foods and diets that are known or suspected of being handled differently by the human and animal intestines. When data are not available for a mixed diet, a weighted average can be calculated from the true digestibility of its constituent protein sources.

Consequently, AA scores as predictors of protein quality must be adjusted for protein digestibility and AA availability. The different scores are the “Chemical amino acid score,” the “Protein Digestibility-Corrected Amino acid Score” (PD-CAAS), and the “Digestible Indispensable Amino Acid Score” (DIAAS) (1, 3, 8, 10). Stable isotope-based methods contribute to accumulate values for true protein and IAA digestibility from human food sources, including animal and plant protein sources. The True ileal digestibility assay is the best currently available approach to assess nitrogen and AA absorption. Digestibility measurements at the ileal level may provide a better measure of AA digestibility, however this may pose significant challenges (9). True ileal AA digestibility is assessed by different invasive or minimally invasive procedures in human, or alternatively in animal (pig or rat) models (9, 11–16).

For both IAA profile and bioavailability, plant protein are most often of lower quality than animal protein (6, 17). Digestibility of protein and IAA from plant protein sources are usually lower than for animal protein sources. The difference is more important when plant proteins are consumed in the form of complex flour or whole grains (treatment, matrix, and antinutritional factors) (2). This is particularly sensitive for younger subjects with higher protein and IAA requirements, i.e., a need for high protein quality. Protein quality also matters in the context of climate change (18). Reduction in diet-associated greenhouse gas emissions involves a shift toward plant-based diets that leads to reduce IAA content, particularly lysine and methionine and a risk to not meet IAA requirements.

## 5 Protein quality and protein synthesis

As mentioned above, the most accurate assessment of protein quality of foods for humans is through clinical studies that measure nitrogen balance (1). Food proteins are fed to a group of individuals and nitrogen losses are determined. However, biological assays in laboratory animals have been used to assess food protein quality, based either on a protein’s ability to support growth in young rats (protein efficiency ratio, PER) or on nitrogen retention (net protein utilization, NPU) (19). The PER and NPU remain useful indices for screening food protein quality and to validate theoretical models based on the AA composition of the target protein. PD-CAAS and DIAAS values in adults for animal and plant protein sources can be compared to the efficiency of nitrogen retention Net Protein Utilization (NPU) (Table 2).

**Table 2:** Protein digestibility, PD-CAAS and Net protein utilization of different protein sources.

	Protein digestibility %	PD-CAAS % (adult)	Limiting AA	Nitrogen/AA retention NPU %
Animal-source	75–99%	>100	–	–
Bovine Milk	94–99	>100	No	75
Meat (beef)	80–99	>100	No	75
Hen egg	80–97	>100	No	72
Plant sources	60–90%	70	–	–
Soy	75–90	86–100	Met+Cys	~70
Pea	70–90	71–78	Met+Cys	~70
Rice	65–85	50–58	Lys	–
Wheat	65–85	46–51	Lys	~60–65

Adapted from Fuller and Tomé (12), Gaudichon et al. (13), Tome (19), Gausseres et al. (20), Evenepoel et al. (21), Bos et al. (22–24), Gaudichon et al. (25), Tomé and Bos (26), Fromentin et al. (27), Oberli et al. (28), and Oberli et al. (29).

AAs are the precursors of protein synthesis in the body. The body proteins and free AAs are in a continuous turnover through protein breakdown and synthesis at an overall rate of about 250–300 g/d (Figure 2). AAs in free form, circulating and present in tissues, are a small fraction of all body AAs (less than 100 g). The 9 IAAs are limiting factor of protein synthesis. The major anabolic factors that influence muscle protein synthesis are contractile activity and feeding. AAs, together with insulin, display an anabolic effect and stimulate muscle protein synthesis (10, 30–33). The ability of a protein source to stimulate protein synthesis have thus been used to assess protein quality. Moreover, among AA, the branched-chain AA (BCAA) have many important physiological roles and of the three BCAA, leucine is most notably a key regulator signaling molecules of muscle protein synthesis (MPS), exerting anabolic effects even in the presence of hyper-aminoacidemia (34).

Protein ingestion induces an increase in muscle protein synthesis (MPS, %/h) measured by stable isotopes method in young men (10, 33). For young adults at rest or with low body exercise 10 g or 20 g of high-quality whey protein result in a rise of MPS of 19 and 52%, respectively, from control 0 g while 40 g do not result in higher stimulation beyond consumption of 20 g. However, in young adults following whole-body exercise 40 g of protein did result in significantly higher MPS rate (35) and results in older adults also indicate a greater MPS response to 40 vs. 20 g whey protein (36). From different studies 40 g protein is consistently 10–20% higher compared to 20 g protein, albeit not always statistically significant (37). Lysine deficiency limits the capacity of wheat protein to induce an increase in MPS. Ingestion in older adult of 35 g wheat protein, deficient in lysine, does not induce an increase in MPS and an increase in MPS was induced by 60 g wheat protein, 35–40 g casein, chicken breast fillet, or lysine-enriched wheat and chickpea protein mixture (38). However, in younger adults an increase in MPS was observed in response to the ingestion of 30 wheat protein (39).

Interestingly, 8 weeks resistance training and intake of 46 g/day high-quality whey (WPC), beef (Beef), or hydrolyzed chicken (Chx) protein after workout improves body composition and muscle performance (38, 40). Lean body mass was significantly increased after 8-weeks resistance training with post workout consumption of a 46 g bolus of WPC, Beef or Chx protein, compared with a control (Maltodextrin) (41).

## 6 Conclusion

Protein requirements relate to the supply of metabolically available nitrogen and IAAs to balance nitrogen and AA losses, to support body protein turnover and synthesis and to maintain the body’s protein pool. Several health outcomes are associated with protein and IAA sufficiency, including growth, body weight, muscle mass and strength, bone health, defenses, and most if not all physiological functions. AA scoring is the preferred approach to evaluate the protein quality. It correlates with other approaches of protein quality (nitrogen retention, protein synthesis, physiological functions). The lower IAA content of certain protein sources is at the origin of the risk of protein deficiency in certain diets. Reference values (data base) on IAA bioavailability of the different protein sources are required.

## Author contributions

JC: Writing – review & editing. DA-M: Writing – review & editing. DT: Writing – review & editing.
